# Milk Protein-Based Nanohydrogels: Current Status and Applications

**DOI:** 10.3390/gels8070432

**Published:** 2022-07-10

**Authors:** Manpreet Kaur, Aarti Bains, Prince Chawla, Rahul Yadav, Anil Kumar, Baskaran Stephen Inbaraj, Kandi Sridhar, Minaxi Sharma

**Affiliations:** 1 Department of Food Technology and Nutrition, Lovely Professional University, Phagwara 144411, Punjab, India; manpreetk.oberoi997@gmail.com; 2 Department of Biotechnology, CT Institute of Pharmaceutical Sciences, South Campus, Jalandhar 144020, Punjab, India; aarti05888@gmail.com; 3 Shoolini Life Sciences Pvt. Ltd., Shoolini University, Solan 173229, Himachal Pradesh, India; yrahul1857@gmail.com (R.Y.); kumaranil_16@rediffmail.com (A.K.); 4 Department of Food Science, Fu Jen Catholic University, New Taipei City 24205, Taiwan; sinbaraj@yahoo.com or; 5 UMR1253, Science et Technologie du Lait et de L’œuf, INRAE, L’Institut Agro Rennes-Angers, 65 Rue de Saint Brieuc, F-35042 Rennes, France; 6 Laboratoire de Chimie Verte et Produits Biobasés, Département Agro Bioscience et Chimie, Haute Ecole Provinciale du Hainaut-Condorcet, 11, Rue de la Sucrerie, 7800 Ath, Belgium

**Keywords:** nanohydrogel, milk proteins, characteristics of milk-protein-based nanohydrogels, food applications

## Abstract

Milk proteins are excellent biomaterials for the modification and formulation of food structures as they have good nutritional value; are biodegradable and biocompatible; are regarded as safe for human consumption; possess valuable physical, chemical, and biological functionalities. Hydrogels are three-dimensional, cross-linked networks of polymers capable of absorbing large amounts of water and biological fluids without dissolving and have attained great attraction from researchers due to their small size and high efficiency. Gelation is the primary technique used to synthesize milk protein nanohydrogels, whereas the denaturation, aggregation, and gelation of proteins are of specific significance toward assembling novel nanostructures such as nanohydrogels with various possible applications. These are synthesized by either chemical cross-linking achieved through covalent bonds or physical cross-linking via noncovalent bonds. Milk-protein-based gelling systems can play a variety of functions such as in food nutrition and health, food engineering and processing, and food safety. Therefore, this review highlights the method to prepare milk protein nanohydrogel and its diverse applications in the food industry.

## 1. Introduction

Hydrophilic gels, or more commonly designated as hydrogels, are three-dimensional networks of polymers that can swell in water and have the potential to retain a high amount within their structure without dissolving [1]. The history of the development of hydrogels is grouped into three generations: first-generation or conventional super-porous hydrogels, and second-generation and third-generation hydrogels [2]. First-generation hydrogels were introduced in 1900 and are termed as a colloidal gel of inorganic salt prepared via the polymerization of water-soluble monomers with multifunctional cross-linkers or with hydrophilic polymers through cross-linking, and they possess a great swelling capacity and good mechanical properties [3]. Second-generation hydrogels came in 1970 and were prepared using environmental factors such as light, pH, heat, and ionic strength and resulted in better control over properties such as drug release, gel formation, biodegradability, and dissolution, e.g., poly(N-iso-propyl acrylamide) [4]. Third-generation hydrogels came in the 1990s and mainly focused on improving mechanical strength and elasticity, and the research is still underway for improving the techno-functional properties of hydrogels [5]. However, recently, hydrogels were used in environmental engineering [6], soft robotics [7], and wastewater treatment [8]. Moreover, hydrogels possess several potential properties such as biodegradability, metabolizability, surface modification, stimulus response, water holding and retaining ability, nonantigenicity, greater stability during storage, easy preparation, as well as controllable size [9]. Due to the unique functional properties, hydrogels discover likely applications in biomedical engineering (drug conveyance, tissue designing, and drug discharge) [10], medical science, agriculture (soil moisturizing, nutrient carrier, and erosion control) [11], textiles, construction [12], diagnostics, regenerative medicines [13], electrical [14], flocculation [15], wastewater treatment, sensors and actuators [16], personal healthcare and hygiene products [17], as well as the food industry (food safety, food nutrition, and food engineering). The covalent and noncovalent interactions such as electrostatic interactions, hydrogen bonds, van der Waals interactions, and intermolecular hydrophobic interactions constitute the chemistry of the hydrogels, and the presence of hydroxyl, amines, carboxyl, ethers, and sulfate groups is responsible for soft and pliable structure [1]. Hydrogels can be synthesized from natural origin (proteins and polysaccharides) as well as synthetic sources (polyvinyl alcohol and polyethylene oxide) [18].

Nanohydrogels, also referred to as aqua gels, are three-dimensional networks of hydrophilic or amphiphilic polymers with diameters less than 100 nm [19]. The word “Nanohydrogel” (NanoGel) was coined to describe the networking and crosslinking of the polyanions and nonionic polymers in the preparation of polynucleotide transport frameworks [20]. Henceforth, these are emerging as creative materials for the development of active packaging in food processing for designing the delivery systems intended for the precise release of nutraceuticals at specific sites of action in the body [21]. Milk is an emulsion produced by the lacteal secretion of mammals. The major components of milk are water, lactose, protein, fat, and minerals. The minor constituents are phospholipids, vitamins, enzymes, sterols, and pigments. The real constituents are milk fat, casein protein, and lactose sugar [22]. The proteins represent the most ‘value-added’ component owing to the budding recognition that bovine milk proteins possess superior dietary and functional characteristics paralleled with other protein sources. Milk comprises around 4–5% of the protein. Milk-based protein nanohydrogels are natural and, hence, categorized as GRAS (Generally Recognized as Safe), inexpensive, nontoxic, and possessing high dietary benefit and exceptional functional, biological, and sensory properties [23]. Milk proteins are made of two significant proteins: caseins and whey proteins. Nanohydrogels can be prepared from caseins, whey proteins (primarily β-lactoglobulin), and their derivatives such as whey protein isolate (WPI) having a protein content of more than 90% and whey protein concentrate (WPC) having a protein content between 50 and 85% on a dry basis [24]. The most popular and successful method to produce whey protein nanohydrogels is the gelation technique that includes both heat and cold-gelation techniques. Gelation of food proteins can be achieved by physical (heat, high pressure), chemical (ionic, pH), and biochemical (enzymes) methods [25]. These are generally utilized as drug delivery frameworks and in the food industry; applications include food nutrition, food safety, food engineering, and food packaging. In this perspective, Li et al. [26] encapsulated epigallocatechin-3-gallate (EGCG), the key catechin in green tea and a powerful antioxidant in nanohydrogels of β-lactoglobulin, and were able to achieve a stable and clear nanosystem at pH 6.4–7.0 and the maximum protection of EGCG antioxidant activity at 85 ℃ and a molar ratio of 1:2 (β-lactoglobulin: EGCG). However, several reports have been undertaken over the last few decades to create hydrogels with stimuli-responsive attributes that correlate to typical hydrogel qualities including porosity, swelling, and physical organization. Therefore, this review article emphasizes milk-protein-based nanohydrogels, the current status of milk-protein-based nanohydrogels in the food industry, along with the methods to formulate milk-protein-based nanohydrogels. In addition, potential applications of nanohydrogel are discussed with schematic diagrams.

## 2. Classification of Hydrogels

**Figure 1:**
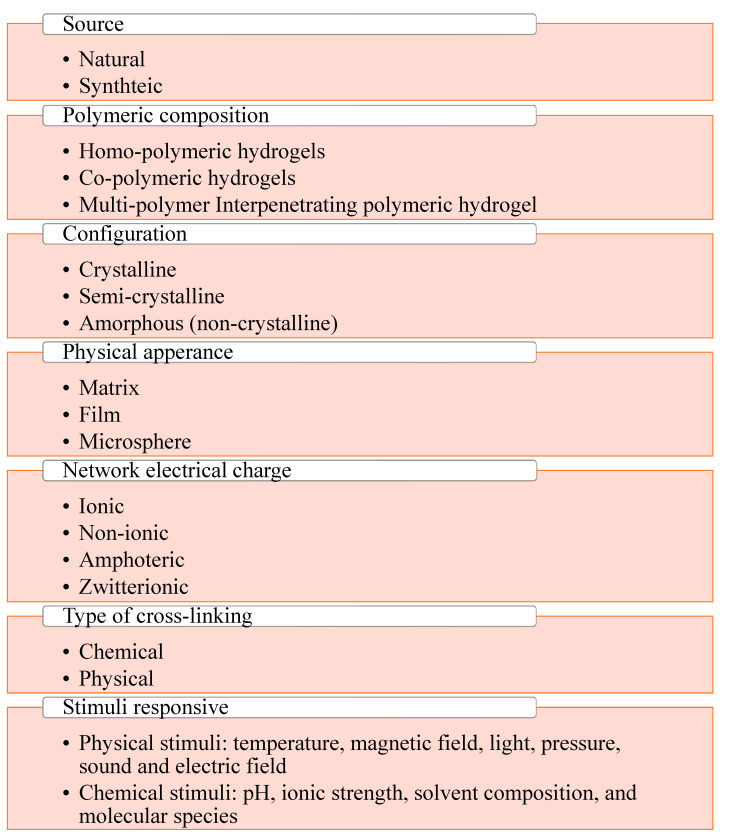
Detailed representation of classification of hydrogels.

Hydrogels are classified based on several characteristics such as cross-linking, stimuli response, preparation source, degradability, electrical charge, physical properties, polymeric composition, configuration, anisotropy, physical appearance, and physical structure [27]. They can be natural or synthetic, where natural hydrogels include polysaccharides (cellulose, alginate, and pullulan) and proteins (milk proteins and soy proteins) and are biocompatible and biodegradable but possess weak stability and mechanical strength [28,29]. However, synthetic hydrogels are prepared through the polymerization of monomers such as polyvinyl alcohol (PVA) and polyacrylamide, and these are stable and have good mechanical strength [30]. The polymer composition can be a homopolymer, copolymer, or multi-polymer interpenetrating polymer. Herein, the homopolymer comprises a single species of monomer, whereas the copolymer comprises two or more diverse polymer species with at least one hydrophilic component, while the multi-polymer interpenetrating polymeric hydrogel comprises two independent cross-linked synthetic and/or natural polymers [31,32]. Furthermore, hydrogels have unique properties such as the ability to be crystalline, semi-crystalline, or amorphous. However, crystalline hydrogels are highly branched polymeric network structures with a definite order of crystallization. In addition, semicrystalline hydrogels contain both crystalline and amorphous regions [33]. Amorphous hydrogels are random network structures at the molecular level. Hydrogels can be prepared in the form of a matrix, film, or microsphere. Similarly, based on their interaction type, a hydrogel can be classified into four categories: ionic (contains anionic and/or cationic groups), nonionic (neutral), amphoteric (contains both acidic and basic groups), and zwitterionic (contains anionic and cationic groups in each structural repeating unit). In this context, several studies have revealed that chemical crosslinked hydrogels are synthesized through covalent bonding, e.g., disulfide formation, and hence have permanent junctions, while physical hydrogels are molecular arrangements prepared through hydrogen bonds, hydrophobic interactions, and van der Waals forces [34]. The response of the hydrogels depends on various physical or chemical conditions. Thus, physical stimuli include temperature, magnetic field, light, pressure, sound, and electric field [35], and chemical stimulations include pH, ionic strength, solvent composition, and molecular species [36]. Milk-protein-based nanohydrogel is natural and considered as stimuli-responsive hydrogels as these are triggered by environmental variables. Milk-protein-based nanohydrogels can be prepared by physical cross-linking as well as chemical cross-linking. In Figure 1, detailed classification of hydrogels are given. 

## 3. Synthesis of Milk-Protein-Based Nanohydrogels

Milk-protein-based nanohydrogels possess high nutritional value as their role in the delivery of bioactive compounds and essential amino acids, specifically sulfur-containing amino acids such as methionine and cysteine [37]. Moreover, the high swelling capability of nanohydrogel provides a proper arrangement for the development of various nanocarriers including nanohydrogel, nanocapsules, nanoemulsion, and nanocomposites. The gels are entirely biodegradable and offer a biological functionality that comprises digestibility conformation and immune system regulation [38]. Therefore, they are completely regarded as safe for human consumption and nontoxic. Milk protein gels especially prepared from β-lactoglobulin, whey protein isolate, and whey protein concentrate can entrap functional components owing to the microscopic size with an enormous internal network for multivalent bioconjugation.

### 3.1. Factors Influencing Gel Fomation

The gel phase transition occurs as a result of a competitive balance between a repulsive force that seeks to expand the polymer network and an attractive force that acts to shrink the network. The electrostatic interaction between polymer charges of the same sort is the most powerful repulsive force, and it may be induced on a gel by injecting ionization into the network. The osmotic pressure created by counter ions contributes to the increasing pressure. Van der Waals contacts, hydrophobic interactions, ion–ion interactions of opposing types, and hydrogen bonding are examples of attractive interactions. The phase change was identified in gels generated by all of the basic forces [39].

The covalent and noncovalent bonds constitute the chemistry of milk protein hydrogels. The main types of noncovalent interactions are electrostatic interactions, hydrogen bonds, van der Waals forces, hydration interactions, and intermolecular hydrophobic interactions [40,41]. Whey protein nanohydrogels are most commonly prepared by the gelation technique [42]. The gel characteristics depend upon various factors such as the protein content, salt concentration, type of ion, pH, ionic strength, the extent of chemical bonding agents utilized, and temperature [43]. Gelation occurs after the unfolding of the original protein structure to prepare a three-dimensional network. Heating is the primary factor behind the formation of food gels containing globular proteins, while other physical (pressure), chemical (acid, ions), and biological (enzymes) forces can be utilized in tandem [44].

Thermal gelation is a phenomenon comprising three stages: (i) primary aggregation via covalent (disulfide bridges) and noncovalent (hydrogen bonds, hydrophobic interactions, and van der Waals interactions) bonds; (ii) secondary aggregation through the joining of primary protein aggregates; (iii) lastly, when the quantity of protein secondary aggregates reaches a threshold concentration, a 3-D network with the potential to entrap water forms [21]. The extent to which proteins denature when heated principally depends upon two factors, intrinsic factors and extrinsic factors. Intrinsic factors comprise solution properties such as pH, protein concentration, and ionic strength, whereas extrinsic factors include heating circumstances such as temperature, heating rate, heating time, and heating technique [45]. New technologies such as dielectric heating (microwave), electric field, and high pressure can also be used in place of heat denaturation [21].

The microwave (MW) is a type of dielectric heating in which an alternating electromagnetic field interacts with polar molecules such as water and ionic species, compelling them to continually realign themselves by reversing an electric field surrounding the food product, causing heat generation [46]. This molecule movement is exceedingly quick due to the high frequency of the field, which can range from 300 to 3000 MHz. According to research by de Pomerai et al. [47], exposure to MW radiation has also been shown to increase protein aggregation, change protein shape without bulk heating, and stimulate the production of particular structures such as amyloid fibrils. Due to its weak penetration capacity, MW heating is frequently claimed to provide uneven heating, which might result in uneven processing. The principal downsides of MW prospecting include nonuniform heating, complexity, expensive equipment costs, difficulties in assuring homogeneity, and a lack of appropriate packing materials. Consequently, this may lead to a number of concerns relating to poor final quality, overheating, and a variety of safety-related issues [48].

The High-Voltage Electric Field (HVEF) is a food processing method that may guarantee product safety while maintaining its characteristics owing to the electric current’s minimally negative effects [49]. The PEF technique has evident advantages because of its low-energy needs and the ability to induce protein structure and functionality alteration without heat side-effects such as the thermal destruction of relevant chemicals. However, heating avoidance is not always practicable, and the nature of the electric pulses (i.e., high voltage) makes full control and automation of the process problematic. Furthermore, the high initial investment cost, as well as the expense of the rigorous maintenance and repair of PEF equipment, prevents widespread industrial application of this technology [50].

Isostatic high pressure (HP) could be employed for food texture engineering due to its influence on the properties of food proteins. At pressures more than 400 MPa (>100 MPa for β-Lg), α-La denaturation is noticed as a decrease in solubility at pH 4.6. However, pressure-induced aggregation resulted in porous gels prone to exudation, as opposed to heat-induced gels with a finely stranded network and good water retention. Pressure denaturation of proteins is a complicated event that is affected by a variety of parameters, including protein structure, pressure range, temperature, pH, and solvent composition [51]. Despite its demonstrated utility in protein functionalization, the extreme complexity of the process, as well as the lack of knowledge of the underlying principles involved, necessitates additional effort to validate HP as a tool in bioscience. The use of technology is also constrained by problems including high equipment prices, high maintenance needs, and scale-up restrictions [50].

### 3.2. Method of Cross-Linking

Gels are formed either by physical cross-linking or chemical cross-linking [52]. Physical hydrogels are also entitled reversible or pseudo-gels, which are networks of heterogenous clusters prepared by molecular entanglements via weak hydrophobic links, ionic interactions, or hydrogen bonding. For instance, a simple thermal process was used to make nanohydrogels from bovine lactoferrin that were resistant to pH (from 3 to 11) and salt (from 0 to 200 mM NaCl) concentration, thus regarded as transporters or functional components in food [21]. Likewise, Relkin et al. [53] used high pressures of 1200 bar to encapsulate α-tocopherol in nanostructures of whey protein dispersals (4% and pH 6.5) and observed a decline in particle charges (to 47 mV) and particle sizes (to 212 nm) and a substantial destabilization of protein structure, though only a 30% vitamin degradation during processing and no degradation during 8 weeks of storage were observed. In another study, the development of lactoferrin nanoparticles by thermal gelation for iron delivery was investigated, where the nanoparticles exhibited an iron-binding efficiency value of ≈20%, were stable to temperature (4–60 °C) and pH (pH 2–11), and achieved a shelf-life of 76 days at 4 °C and, hence, presented a pH-dependent behavior [54]. Caffeine (hydrophilic) and curcumin (lipophilic) were proficiently encapsulated in lactoferrin-glycomacropeptide (LF-GMP) nanohydrogels with great encapsulation efficiencies (>90%) by the thermal gelation technique [55]. The release mechanism of these bioactive compounds at different pH was performed and a pH-dependent release profile was observed. Similarly, thermally induced whey protein isolated hydrogels have also been used to encapsulate anthocyanin-rich bilberry extracts, demonstrating a Fickian diffusion-based release mechanism of bioactive phenolic compounds in simulated gastric juices [56].

On the other hand, chemical hydrogels also termed as irreversible or permanent gels are polymeric networks prepared by covalent bonding at definite sites and, thus, can absorb water, swell, and hold until equilibrium is attained [57]. Chemically cross-linked hydrogels can form a superior hydrogel in terms of swelling and gelling capacity. In a study conducted by Donato et al. [58], the influence of mono- and divalent salts (NaCl and CaCl_2_, respectively) concentration on heat-precipitated bovine serum albumin gels at pH 7.0 was determined, and it was concluded that repulsive forces were decreased at excessive salt ranges and CaCl_2_ changed into a greater green color than NaCl in identical ionic energy upon decreasing those interactions.

### 3.3. Type of Gel Formed

Globular proteins may form both hot and cold-set gels [59], and the production of hot-set gels is initiated by the aggregation of the unfolded protein moieties of an intermolecular β-sheet network [60]. As these gels have a high strength, consistent structure, and ability to form a gel at ambient temperature [61], cold-set gels are exceptional for formulating functional meals with better control over shape, structure, and texture along with targeted delivery [62]. The formation of cold-set gels occurs in two stages. The first stage is to heat the protein at neutral pH (above isoelectric point), below the protein gelling ability, and at low ionic strength, which causes protein denaturation and poor folding. The second stage is acidification to approach the isoelectric pH of protein (acid-induced cold gelation) or salt supplementation (salt-induced cold gelation) to generate cross-linkages among protein clusters and reduce the inter-protein repulsion [63]. For the cold gelation of globular proteins, calcium chloride and sodium chloride are utilized [64]. Alternatively, cations such as Fe^2+^ [61] and Mg^2+^ [65] were employed to create whey protein cold-set gels having better functional and nutritional value. In another study, the characteristics of ion-encased whey protein cold-set gels can be influenced by the heat treatment of protein solution. Martin et al. [61] investigated the impact of different heat treatments (mild, intermediate, and severe) on the iron encapsulation effectiveness of a whey protein cold-set gel. In comparison to mild (85 °C, 30 min, and pH 7.0) and severe (pH 2.0 and 80 °C for extended period), cold-set gel synthesized from whey protein bearing intermediate heating conditions (85 °C, 3 h, and pH 3.35) exhibited a higher iron release at natural pH owing to the formation of different structural entities as a function of heating conditions. At acidic pH, however, there was no discernible difference in iron release among the samples.

Casein protein is present as a calcium caseinate-phosphate complex [66]. It is in a colloidal state and forms more than 80% of the complete protein in milk [67]. Transglutaminase can covalently join glutamine deposits with the remaining lysine due to the low degree of secondary and tertiary arrangements, converting casein micelles to nanogel particles [68]. However, casein hydrogels can also be made using genipin, a naturally derived chemical that can join amino groups of lysine, hydroxylysine, or arginine residues in various polypeptide chains utilizing monomeric or oligomeric cross-linkers. The encapsulation of bioactive compounds such as probiotic organisms [69], fats and oils [59], and vitamin B12 is the most promising application of casein hydrogels [70].

**Figure 2:**
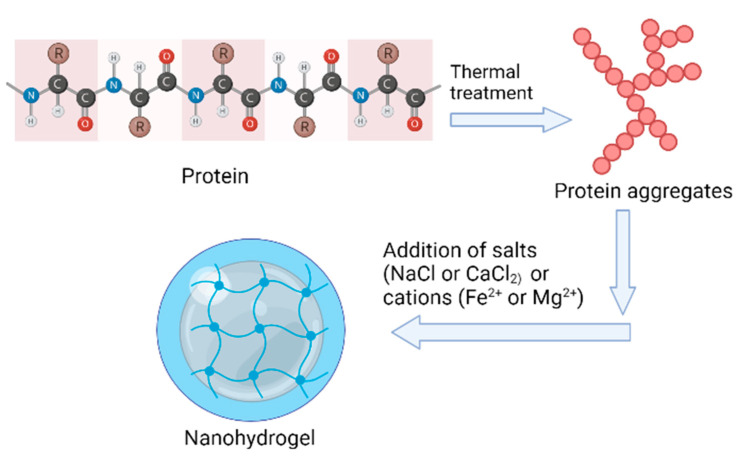
Schematic representation of synthesis of milk-protein-based nanohydrogel.

Consequently, proteins can be blended with polysaccharides effectively to form hydrogels to be used for encapsulation and delivery of nutrients. In this context, Ozel et al. [71] blended polysaccharides (xanthan gum, pectin, and gum tragacanth) with heat-set whey protein and utilized it to encapsulate the dark carrot extricate. Likewise, for encapsulation and delivery of hydrophobic nutraceuticals such as w-3 fatty acids, Salimen and Weiss [72] created a stable nanohydrogel produced from a compound of protein-polysaccharide (i.e., β-Lg-Pectin) and achieved stability against oxidation during storage (Figure 2). 

## 4. Characterization of Milk Protein Hydrogels

### 4.1. Fourier Transform Infrared Spectroscopy (FTIR)

**Figure 3:**
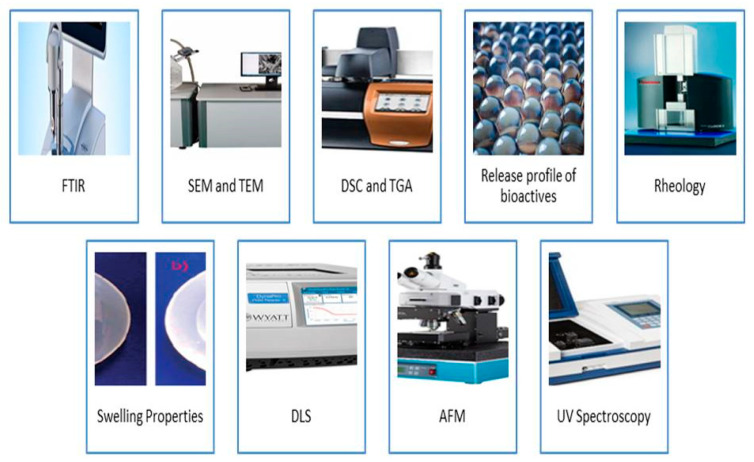
Schematic representation of characterization of milk protein nanohydrogels.

Characterization of formulated gels based on various techniques is an important feature and in Figure 3 all the instrumental techniques are revealed. FTIR is an analytical method performed to investigate the presence of chemical bonds or functional groups in the hydrogel by the use of an infrared absorption spectrum [73]. The presence of the amide bond in the protein or peptide-based hydrogel can be identified using FTIR. The results of FTIR as concluded by several authors demonstrated that hydrogel prepared by the protein-polysaccharide complex consists of C = O, C–O, O–H, C–C, C–H, C–N, and N–H. For instance, curcumin- and caffeine-encapsulated Lactoferrin-Glycomacropeptide (Lf-GMP) nanohydrogel showed characteristic bands of caffeine in Lf-GMP as peaks at 1707 cm, 974, and 1359 cm^−1^ corresponding to C = C, C–C, and C–H stretching [74]. Additionally, it is possible to assure the encapsulation of caffeine in Lf-GMP nanohydrogels owing to changes in the amide I band (1700–1600 cm^−1^), mainly C = O stretching, and amide II band (1600–1500 cm^−1^), C = N stretching coupled with the N–H bending mode, signifying a hydrophilic interaction between the caffeine and Lf-GMP nano hydrogel; meanwhile, the major groups involved are C = O, C–N, and N–H [75,76]. Consequently, several gums have shown great complex binding with milk protein. In this context, Pilevaran et al. [77] synthesized the hydrogel using whey protein and xanthan gum. In their study, different amounts of gums were used for the synthesis of the hydrogel. However, the FTIR test clearly showed that the peak observed at 1649 cm^−1^ was related to the C–N stretching of the amide ΙΙ band and the C–N–H in-plane bending and correlated to C–H bending. The two strong peaks perceived at 1073 and 1155 cm^−1^ relate to saccharides.

### 4.2. Morphological Characterization of Nanohydrogels

Scanning electron microscopy (SEM) is used to describe the microstructure of the surface morphology of the polymer. The porosity of the hydrogel, three-dimensional network, and surface morphology are analyzed using scanning electron microscopy (SEM). SEM is the most commonly used technique for morphological studies of hydrogels irrespective of their composition [78,79]. Hamcerencu et al. [80] conducted a study on whey protein concentrate-Xanthan gum (WPC-XG) hydrogel and showed spherical and large aggregates of protein with high porosity and various sizes, whereas WPC hydrogel showed a more compact structure with lower porosity in comparison with WPC–XG hydrogels.

Similarly, Transmission Electron Microscopy (TEM) was used to understand the morphology of Lf-GMP nanohydrogels. TEM images show that Lf-GMP nanohydrogels with bioactive compounds encapsulated maintain the spherical shape with a solid dense structure of sizes around 120 nm and 125 nm for caffeine and curcumin, respectively.

### 4.3. Differential Scanning Calorimetry

The thermal stability change in the structural strength of protein-based nanohydrogels with temperature is measured via a differential scanning calorimeter (DSC) or thermogravimetric analysis (TGA). The relative change in the structure of the prepared hydrogel with temperature is compared with the standard protein hydrogel. Parameters such as glass transition temperature and melting point are important characteristics to reveal the thermoplastic behavior of protein nanohydrogels determined by a DSC or TGA [81,82]. In the DSC technique, the difference in the quantity of heat required to raise the temperature of a sample compared to a reference is measured as a function of temperature. A single endothermic peak was detected for all the hydrogels. Barsett et al. [83] performed DSC on Lf-GMP hydrogel and observed the highest peak temperature at 104 °C, the onset temperature at 61.5 °C, and the end set temperature at 130 °C related to XG 0.01%; the lowest peak temperature at 98 °C, the onset temperature at 64.5 °C, and the end set temperature at 128 °C related to WPC hydrogel. On the other hand, the WPC–XG composite hydrogel at 0.3 and 0.6% of XG had more thermal stability. In addition, DSC revealed that XG and WPC interacted and promoted the formation of a 3D network structure.

### 4.4. Release Profile of Bioactives

In a study performed by Vesely et al. [84], the experiments of bioactive compounds released from Lf-GMP nanohydrogels were conducted at 37 °C at two different pH values: 2 and 7. These conditions were used to simulate the release mechanisms of these bioactive compounds when subjected to digestion in the human gastrointestinal system. The release profile of caffeine from Lf-GMP nanohydrogels revealed that at pH 2, a higher amount of caffeine was released from Lf-GMP nanohydrogels and, thus, it was concluded that bioactive molecules are on the surface of the carrier and are released faster than if they were entrapped. Unlike caffeine, curcumin release from Lf-GMP nanohydrogels is pH-dependent, as, at pH 2, there is a clear release profile of curcumin from nanohydrogel, whereas, at pH 7, no curcumin was released and, hence, it was concluded that curcumin is more triggered under acidic conditions than under a basic environment [85].

### 4.5. Rheological Characterization of Nanohydrogels

Rheological evaluation of the protein nanohydrogels demonstrates the impact of environmental variables such as pH, temperature, enzyme concentration, and ion concentration on rheological characteristic parameters such as viscoelastic characteristics, gelation time, gel strength, yield-strain, and various properties such as stiffness, gelation kinetics, viscoelastic regions, relative liquid–solid properties, and relaxation time scale [86]. Oscillation rheology is used to study the rheological behavior. In oscillation rheology, sinusoidal shear is applied and the resulting stress is measured as a function of time [87].

### 4.6. Determination of Swelling Properties

Hydrogels are cross-linked polymer networks swollen in a liquid state. The absorbed liquid behaves as a selective filter and allows free diffusion of few solute molecules, whereas the polymer network acts as a matrix to hold liquid together. Hydrogels can soak up to a thousand times their dry weight. It can be estimated by measuring the dry weight and the swollen state weight and calculating either the water uptake or a volume of adsorbed solvent [88]. The assessment of swelling also acts a measure for other hydrogel properties such as cross-linking degree, mechanical properties, and degradation rate:

Water uptake (in %) = swollen weight-dry weight/dry weight∗100

Volume of adsorbed solvent (in %) = swollen weight-dry weight/water density∗100. Hydrogels immersed in the aqueous medium absorb water due to the osmotic pressure difference between the gel network and surrounding environment [89]. In a study conducted by Taskooh [90] on freeze-dried hydrogel samples placed in 25 mL of buffer solution (pH 7, 0.1 M) at 21 °C, the swelling ratio increased from 15.5 ± 6.3 to 20.34 ± 5.75 (*p* < 0.01) with varying iron concentration from 10 mM to 70 mM. By increasing the cross-linker (Fe) concentration, the density of cross-links was enhanced so that the water absorption capacity and swelling ratio improved.

### 4.7. Dynamic Light Scattering (DLS)

DLS is important to measure size changes for micro- as well as nanosized polymer gels. Nanoscale aggregation phenomena during the initial stages’ steps of milk protein aggregation as affected by the thermal treatment are assessed by DLS. In a study conducted by Silva et al. [91], DLS was performed on the casein hydrogels prepared using 5 mM, 10 mM, and 20 mM in a casein dispersion of 2.5%, and it showed a decrease in the hydrodynamic diameter (Dh) of the casein micelles as a function of genipin concentration. In the control sample (without GP), it was 179 nm, whereas it was 160 nm with the 20 mM sample. The dispersion viscosities also decreased when higher genipin concentrations were tested assuming values from 1.655 mPa.s^−1^ (control) to 1.441 mPas.s^−1^ (20 mM genipin).

### 4.8. Atomic Force Microscopy (AFM)

AFM has been used to determine biomolecule morphological features such as distinct molecular shapes and surface coverage. It is also a nondestructive imaging approach that is commonly used to investigate the architecture of biological systems, such as proteins. This was another microscopic approach employed in the investigation of casein hydrogels carrying insulin performed by Alibolandi et al. [92] and is an appropriate way for evaluating the surface of hydrogel samples. The linkages between molecules and the loops were the prominent and concave components, respectively. The tapping-mode AFM pictures of hydrogel and its height profile were illustrated and validated the creation of a three-dimensional network structure in the casein hydrogel.

### 4.9. UV Spectroscopy

Protein hydrogels absorb small wavelengths in the UV region of the spectrum presenting an absorption peak at 280 nm [93] and low absorption in the visible and near-infrared. Whey protein hydrogel was prepared using copper as a cross-linking agent (CuSo_4_) and the absorbance was measured. The use of whey proteins led to an increase in the absorbance of the film due to changes in the structure of the hydrogel as a higher absorption (smaller transmittance) was obtained by increasing the Cu^2+^ films.

## 5. Applications of Milk Protein-Based Nanohydrogels

**Figure 4:**
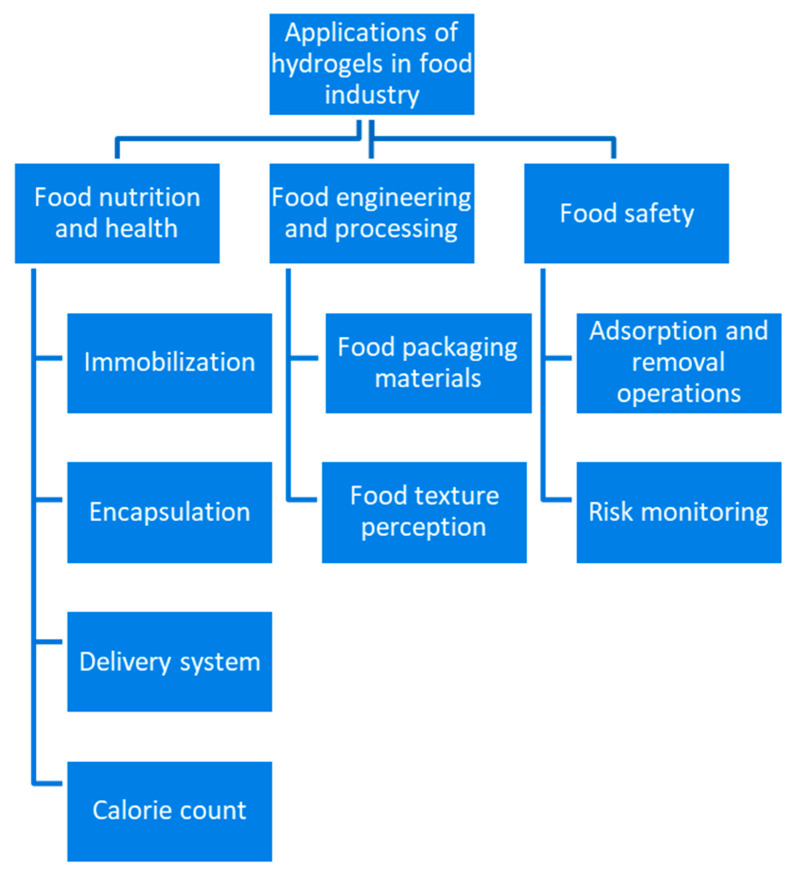
Applications of nanohydrogels in the food industry.

Nanotechnology refers to the study of particles/objects/materials/matter with sizes ranging from 1 to 100 nm [94]. Herein, Figure 4 is revealing all the applications which are directly or indirectly related to the milk protein-based nanohydrogels. In the food industry, nanotechnology is applied to all the sectors such as food processing (encapsulation and defined discharge of nutraceuticals or food additives with improved bioavailability), food packaging (active and smart packaging systems for better food safety and biosecurity), food safety assurance (detection of contaminants or foreign objects or undesirable microorganisms using nanosensors, risk monitoring, adsorption, and removal operations), and food quality control (calorie count and food texture perception) [95].

Micronutrients such as minerals, vitamins, and nutraceuticals are more explicitly targeted by nanodelivery in the food industry [96]. However, significant challenges arise in the absorption and distribution of micronutrients through diet such as poor chemical stability, less oral bioavailability, poor solubility, and undesirable sensory attributes. These constraints must be overcome by consolidated logic and designing a methodology to be more specific; the job of nanodelivery for micronutrients includes improved gastrointestinal stability, security against oxidation, controlled and designated discharge, enhanced bioavailability as well as bioactivity, and assurance of the active compound during food handling stockpiling and circulation [97].

The ideal characteristics of a delivery system include properties such as (i) the ability to deliver active compounds exactly at the target place, (ii) efficiency of maintaining the active compound at desired levels during storage, and (iii) ensuring availability at a specific rate and targeted time [98]. All these features are fulfilled by the protein-based nanohydrogels, particularly the whey-protein-based nanohydrogels with greater efficiency, and hence, these are considered as an ultimate drug delivery arrangement due to their high stability, optimal drug loading ability, a multi-response approach aiming at a specific position, biological consistency, and reaction to a variety of external environmental stimuli [99].

Whey-protein-based hydrogels can entrap both the hydrophilic (e.g., caffeine) and lipophilic (e.g., curcumin) compounds without affecting their activity much, thus contributing to the development of novel functional foods [100]. Owing to their biocompatibility feature, hydrogels are regarded as a blessing in tissue engineering and medicine delivery [101]. Moreover, when nanoparticles are successfully integrated into hydrogels, they can be targeted specifically to tumor locations. Nanohydrogels play an essential role in the delivery of nanomedicine through the epitome of biomolecules [102]. Table 1 lists the applications of whey-protein-based nanohydrogels in the food industry.

Whey proteins are extensively employed in the food sector due to their exceptional functional characteristics such as emulsifying, foaming, gelling, as well as solubility [103]. The functional properties of proteins directly play a key role in organoleptic (color and flavor), kinesthetic (mouthfeel, smoothness, and texture), as well as textural (chewiness, elasticity, adhesiveness, and cohesiveness) features [104]. In other words, owing to their inherent viscoelastic properties, nanohydrogels can act as emulsifying and foaming agents in food stabilization and form firm nanocomplexes with polysaccharides, and hence, they are considered imperative aspects in the preparation of nanohydrogels for applications in the food industry [105].

The capacity of globular proteins to form gels under cold circumstances has recently received attention due to its applicability in innovative food and nonfood areas [106]. Apart from calcium and sodium ions, iron may be employed as an effective delivery vehicle via protein gelation as the amino acids improve iron ion bioavailability; hence, the concentration of iron influences the structure of the gels as, at low iron concentrations, gels are prepared as filamentous microstructures due to hydrophobic interactions, whereas the gels set at high iron ion concentrations have random spherical aggregates with significant van der Waals forces [107]. Filamentous nanohydrogels make up an outstanding matrix for the transportation of iron, thus stimulating its absorption and permitting the development of novel functional foods with targeted mineral deficiency [108].

As hydrogels possess a three-dimensional porous structure, hydrogels may absorb up to many thousands of times their dry weight, making them an ideal encapsulation technology for water-soluble components [21]. Immobilization (enzyme, cell, and microorganism) also takes place within the hydrogel network and on the hydrogel’s surface, where the surface and internal structure characteristics of hydrogels have a significant impact on their immobilization capacity [109]. Hydrogels are excellent for use in delivery systems due to their unique ability to absorb and store significant volumes of water or biological fluids inside their three-dimensional network [110].

The main role of the hydrogel in food packaging is to control humidity inside the package as it lowers the water activity and retards the growth of spoilage microorganisms on foods [111]. Hydrogels possess great antimicrobial activity when incorporated with different antimicrobial compounds such as nanoparticles (silver) or bioactive compounds [112]. In addition to this, hydrogels show potential applications in smart food packaging as they either act as an indicator regarding the freshness of the food or detect the presence of contaminants [113]. Hydrogels have become important in food safety as they act as sensor or signal probes to detect hazards in food. Natural hydrogels are being investigated for the detection of biological hazards in foods. Hydrogels can be used to improve the texture or mouthfeel (elasticity, hardness, and chewiness) of the food owing to their soft texture [114]. Hydrogels play a vital role in lowering the calorie content of the food by either enhancing satiety or by reducing intake [115]. Wu et al. [116] fabricated a hydrogel using protein-dietary fiber, which acted as healthier replacements for starch granules. Hydrogels act as an efficient platform for adsorption and removal operations owing to their high-water retention capacity, high porosity, and reusability [117].

**Table 1:** Applications of whey-protein-based nanohydrogels in the food industry.

Gel Components	Carrier/Cargo	Gelation Technique	Application	References
Whey protein isolate/lauric acid	Echium oil	Physical self-assembly	Encapsulation/delivery	[118]
Whey protein concentrate/Pectin	D-Limonene	Heating	Encapsulation/delivery	[119]
β-Lactoglobulin nanoparticles	Caffeine	Thermal gelation	Delivery	[120]
Whey protein isolate	Iron	Salt-induced gelation	Fortification of food systems and site-specific delivery of iron	[121]
β-lactoglobulin/alginate	Quercetin	Heating	Encapsulation/ Delivery	[122]
β-Lactoglobulin/Chlorogenic acid	Epigallocatechin-3-gallate	Gelation	Encapsulation/delivery	[123]
Whey protein isolate and polysaccharides	Black carrot extract	Heating	Organized delivery conditions for bioactive agents	[71]
Whey protein isolate	Caffeine	Heating	Delivery of nutraceuticals	[124]
Whey protein isolate and niosomes	α-tocopherol	Acid-induced gelation	Intestinal delivery and improved bioavailability of α-tocopherol	[125]
α-Lactalbumin	Curcumin	Temperature-induced gelation	Delivery of bioactive therapeutic agent helping treat various human diseases	[126]
Whey protein concentrate	Phytosterols	Gelation	Encapsulation/delivery	[127]
Lactoferrin and Glycomacropeptide	Curcumin and caffeine	Thermal gelation	Bioactive compound carrier	[55]
β-Lactoglobulin	Vitamin B2	Gelation	Encapsulation/delivery	[128]
Whey protein concentrate	Folic acid	Electrospray particles	Encapsulation of bioactive compounds	[129]
Sodium caseinate, whey protein isolate, and soy protein isolate	β-Lactoglobulin	Heating	Encapsulation/delivery	[130]
Whey Protein Isolate/Pectin	Anthocyanin	Heating	Encapsulation/delivery	[131]
Bovine serum albumin and Polyethylene glycol	5-Fluorouracil	Heating	Injectable drug transport medium	[132]
β-Lactoglobulin/Zein	Tangerine	Gelation	Encapsulation/delivery	[133]
β-Lactoglobulin/Dextran	β-carotene	Temperature-induced gelation	Encapsulation/delivery	[134]
Whey protein isolate	Fe^2+^ and ascorbate	Salt-induced gelation	Increase in Fe^2+^ bioavailability, formulation development for fortification of food with iron	[61]
Whey protein concentrate	α-Tocopherol	Heating and high pressure	Encapsulation/delivery	[53]
Whey protein isolate	Zinc	Heating and ethanol desolvation	Encapsulation/delivery	[135]
β-Lactoglobulin	Catechin	Heating	Encapsulation/delivery	[136]
β-Lactoglobulin	Epigallocatechin-3-gallate	Thermal gelation	Encapsulation/delivery	[26]
Whey protein isolate	Bilberry extract	Heating	Whey-protein-based acidic gels are used for the encapsulation and stabilization of anthocyanin-rich bilberry extract	[137]
β-Lactoglobulin	Fe^2+^	Salt-induced gelation	Development of filamentous gel matrix for intestinal delivery of iron	[138]
Whey protein concentrate, alginate	Caffeine	Heating	Hydrogels resistant to proteolytic enzymes in the stomach	[139]
β-Lactoglobulin	Fe^2+^	Salt-induced gelation	Increase in the bioavailability of iron ion	[140]
β-Lactoglobulin and alginate	α-tocopherol	Salt-induced gelation	Intestinal delivery and bioavailability improvement of α-tocopherol	[141]
Whey protein isolate	Ethyl hexanoate	Heating and ethanol desolvation	Encapsulation/delivery	[142]
Whey protein isolate and tara gum	Magnesium	Salt-induced gelation	Preparation of gels with a wide range of textural qualities for use in the food industry	[65]
Whey protein concentrate and honey	-	Heating	In the formulation of desserts such as flans, cakes, and tart fillings	[143]
Bovine serum albumin and acrylamide	Salicylic acid or sodium benzoate	Copolymerization of vinylated bovine serum albumin and acrylamide	Constant drug discharge agent for substances binding with albumin	[144]
Whey protein isolate		Thermal gelation	Structuring	[145]
Methacrylate-derivatized bovine serum albumin and methacrylic acid sodium salt	Diflunisal and β-propranolol	Free radical polymerization	Oral drug carriers with a high degree of swelling and increased water affinity	[146]
β-lactoglobulin and low methoxy pectin	ω-3 fatty acids	Physical self-assembly	Encapsulation/delivery	[147]
β-Lactoglobulin	α-tocopherol	Temperature-induced gelation	Encapsulation/delivery	[141]
β-Lactoglobulin	Curcumin	Temperature-induced gelation	Encapsulation/delivery	[148]

## 6. Conclusions

Protein nanohydrogels are being investigated for use in a variety of fields, including the delivery of chemicals. The nature of the component protein, other conjugated polymeric systems, pH, temperature, types of ionic structure, oxidative-redox conditions, and ionic strength influences the characteristics of nanohydrogels. The release profile of bioactive chemicals from the hydrogel may be altered by fine-tuning the aforementioned properties. Nowadays, protein nanohydrogels are being investigated as adaptable vaccine delivery vehicles to elicit a vigorous immune cell reaction. Protein hydrogels can shield cells, peptides, biomolecules, and pharmaceuticals from a hostile environment. Nanoscale delivery devices that contain active compounds are effective in protecting them from deterioration throughout processing, transport, and storage. Furthermore, nanoencapsulation can conceal off-odors and flavors of bioactives and ease food integration. Aside from these advantages, nanoencapsulation assists in the regulated, prolonged release of the nutrient at the active site, hence increasing its bioavailability. Whey proteins are incredibly adaptable, nutritious, and cost-effective dietary items that may be employed as rich matrices to build a variety of nanostructures in diverse ways owing to their reactivity to diverse environmental variables (e.g., pH, temperature, electric field, and ionic strength). Although whey protein nanostructures have the potential to be useful in a wide range of consumer food products, significant challenges remain to be overcome, including their large-scale production, their stability in challenging processing and storage environments, physical and chemical interactions between foods encapsulating sensitive molecules, their sturdiness and flexibility in the gastrointestinal tract, and the sense of awareness and satisfaction among consumers. At present, a better understanding of the action mechanism of the milk-protein-based nanohydrogels in the gastrointestinal tract will help in the optimization and expansion of nanotechnology in the drug delivery sector. In order to attract consumers, an in-depth study of the bioavailability, permeability, and toxicity needs to be addressed. Hence, it is crucial to develop predictive and validated toxicological tests to identify potential risks to humans, which could include oxidative damage, lesions of the kidney and liver, inflammation of the gastrointestinal tract, and cancer. Overall, the expansion of milk protein nanosystems for oral delivery of bioactive compounds has sparked research, positioning itself not only as a budding solution to food industry problems but also as an inventive tool for pharmaceutical uses. As a result, the potential of nanotechnology for solving various challenges is recognized by the US-FDA and it is providing support to overcome these nanotechnology-based challenges.

## Data Availability

Data sharing is not applicable to this article.
